# Acute D-Serine Co-Agonism of β-Cell NMDA Receptors Potentiates Glucose-Stimulated Insulin Secretion and Excitatory β-Cell Membrane Activity

**DOI:** 10.3390/cells10010093

**Published:** 2021-01-07

**Authors:** Amber Lockridge, Eric Gustafson, Alicia Wong, Robert F. Miller, Emilyn U. Alejandro

**Affiliations:** 1 Department of Integrative Biology and Physiology, University of Minnesota, Minneapolis, MN 55455, USA; lockr008@umn.edu (A.L.); gusta080@umn.edu (E.G.); wongx615@umn.edu (A.W.); 2 Department of Neuroscience, University of Minnesota, Minneapolis, MN 55455, USA; rfm@umn.edu

**Keywords:** β-cell, D-serine, glucose homeostasis, insulin secretion, mice, Grin1, NMDA receptor

## Abstract

Insulin-secreting pancreatic β-cells express proteins characteristic of D-serine regulated synapses, but the acute effect of D-serine co-agonism on its presumptive β-cell target, N-methyl D-aspartate receptors (NMDARs), is unclear. We used multiple models to evaluate glucose homeostasis and insulin secretion in mice with a systemic increase in D-serine (intraperitoneal injection or DAAO mutants without D-serine catabolism) or tissue-specific loss of Grin1-encoded GluN1, the D-serine binding NMDAR subunit. We also investigated the effects of D-serine ± NMDA on glucose-stimulated insulin secretion (GSIS) and β-cell depolarizing membrane oscillations, using perforated patch electrophysiology, in β-cell-containing primary isolated mouse islets. In vivo models of elevated D-serine correlated to improved blood glucose and insulin levels. In vitro, D-serine potentiated GSIS and β-cell membrane excitation, dependent on NMDAR activating conditions including GluN1 expression (co-agonist target), simultaneous NMDA (agonist), and elevated glucose (depolarization). Pancreatic GluN1-loss females were glucose intolerant and GSIS was depressed in islets from younger, but not older, βGrin1 KO mice. Thus, D-serine is capable of acute antidiabetic effects in mice and potentiates insulin secretion through excitatory β-cell NMDAR co-agonism but strain-dependent shifts in potency and age/sex-specific Grin1-loss phenotypes suggest that context is critical to the interpretation of data on the role of D-serine and NMDARs in β-cell function.

## 1. Introduction

D-serine is a non-proteinogenic amino acid with a critical role in the central nervous system (CNS) as a regulator of neural development, plasticity, excitability, and viability but its potential role in peripheral tissues has received less attention. We previously showed that both human and mouse pancreatic islets express the D-serine synthetic enzyme serine racemase (Srr) [1], for which genetic polymorphisms have been correlated to gestational diabetes [2], type II diabetes [3], and the therapeutic efficacy of the antidiabetic drug metformin [4]. Our work and other recent studies have demonstrated changes in glucose homeostasis and insulin secretion in association with Srr loss [1], chronic D-serine supplementation [5], and the inhibition or tissue-specific loss of the D-serine targeted N-methyl D-aspartate receptors (NMDARs) [6]. However, these changes appear to be unusually context-specific with, for example, both hyperinsulinemic and hypoinsulinemic effects of D-serine drinking in male mice, dependent on dose [5]. This is consistent with CNS literature on D-serine-mediated systems, which are highly plastic and require precise activating conditions, the effect of which may be bi-directional depending on factors like developmental time window [7,8], target composition and localization [9,10] and repeated exposure [11]. Therefore, while excellent work in genetically-static and chronic administration models has converged on a novel role for D-serine in the regulation of glucose homeostasis, the absence of a thorough characterization of D-serine’s acute effects, particularly on glucose-stimulated β-cell insulin secretion, remains a significant barrier to understanding that role.

The in vivo target of D-serine is the NMDAR, a non-specific cation channel whose subunits have been detected at both the transcript and protein level in multi-species β-cell lines and in primary isolated islets [6,12,13,14,15,16,17], which are predominantly composed of β-cells. In neurons, NMDARs are a critical molecular determinant of cellular memory by adjusting the excitability threshold, and thus the stimulus-excitation coupling, of post-synaptic membranes (reviewed e.g. in [18]). In order to activate, classical NMDARs (containing GluN1 and GluN2 subunits) require multiple simultaneous inputs: a local depolarization to expel a Mg^2+^ pore-block, the binding of an agonist like L-glutamate and the binding of a co-agonist (i.e., D-serine or glycine). Critically, these NMDARs will not activate in the absence of any one of these conditions and in several cell types, D-serine is a far more potent channel activator than glycine [11,19,20]. This may help to explain why studies attempting to investigate β-cell NMDAR activity and function have variously associated it with insulin secretion that is increased [12,13], decreased [6,15,17] or unchanged [14]; membrane currents that are depolarizing [12,14,16], hyperpolarizing [6,16], or both [16]; and elevated [12,14,16,21] or unchanged [15] intracellular Ca^2+^ ([Ca^2+^]_i_). So while several recent papers have developed a narrative around a functionally inhibitory role for β-cell NMDAR activity, divergent results in the broader literature, the near exclusive use of glycine as an exogenous co-agonist, and the interpretation of data based on uncertain activation conditions make it difficult to project these findings onto a hypothesis of how D-serine may regulate β-cell function and glucose homeostasis in an acute timeframe.

In the current study, we characterize several models in which systemic elevations in D-serine were associated with improved glucose tolerance and hyperinsulinemia in mice, including a genetic loss of the D-serine catabolic enzyme D-amino acid oxidase (DAAO) and following an intraperitoneal (i.p.) injection of 1–3 g/kg of D-serine in multiple wildtype (WT) mouse strains. We found that D-serine also potentiated in vitro glucose-stimulated insulin secretion (GSIS) of mouse islets and depolarized the membranes of individual β-cells, but this was dependent on the expression of β-cell NMDARs and the inclusion of a receptor-specific glutamate analog (NMDA). Furthermore, NMDAR loss (Grin1/GluN1 KO) was associated with impaired islet GSIS and glucose intolerance in some models. However, these results were all context-dependent with strain-based shifts in D-serine potency, as well as age, diet, and sex-based differences in the in vitro and in vivo phenotypes of β-cell and pancreatic Grin1 KO mice. We found evidence of increased expression of the “inhibitory” Grin3a NMDAR subunit in C57 vs. FVB tissues, which might contribute to the reduced D-serine sensitivity in the former strain but a full accounting of cellular mechanisms underlying the diversity of responses to β-cell NMDAR manipulations in the literature remains to be done. Nevertheless, our data demonstrate the potential for D-serine co-agonism, under proper NMDAR activating conditions, to stimulate β-cell excitability and potentiate insulin release, similar to its classical regulation of excitatory neurotransmission in the CNS.

## 2. Materials and Methods

### 2.1. Experimental Subjects

As previously described [22,23], D-amino acid oxidase (DAAO) mutant mice (DAAO^−/−^) and ddY background controls were originally obtained from the Konno lab [24], then bred for experiments at the University of Minnesota. C57Bl/6J (strain 000664) and FVB (strain 001800) male and female mice were purchased from Jackson labs and rested for at least 1 week before use. Grin1 loss models, on a C57Bl/6J background, were generated by breeding Rip-cre or Pdx1-cre containing male mice (a gift from Dr. Pedro Herrera, University of Geneva, Switzerland) with Grin1 flox/flox (f/f) female mice from Jackson (strain 005246). Cre-driver efficiency and specificity was confirmed by crossing in a single CAG-ZsGreen allele (see also [25]). All mice were group housed with ad libitum access to food and water on a 14:10 light cycle. Mice were fed on standard rodent chow or a 60% kcal high fat diet (HFD) (Research Diets, D12492). Unless otherwise indicated, all mice tested were 2–4 months old. A combination of male and female islets was used for all Grin1 and D-serine/NMDA GSIS and electrophysiology experiments (shown in diamonds). For all other assessments, the sex of the subjects is indicated in the figure legend. Cre-negative (Grin1 f/f or Grin1 f/+) mice were used as WT controls against Grin1 loss models.

### 2.2. In Vivo Mouse Experiments

**Figure 1:**
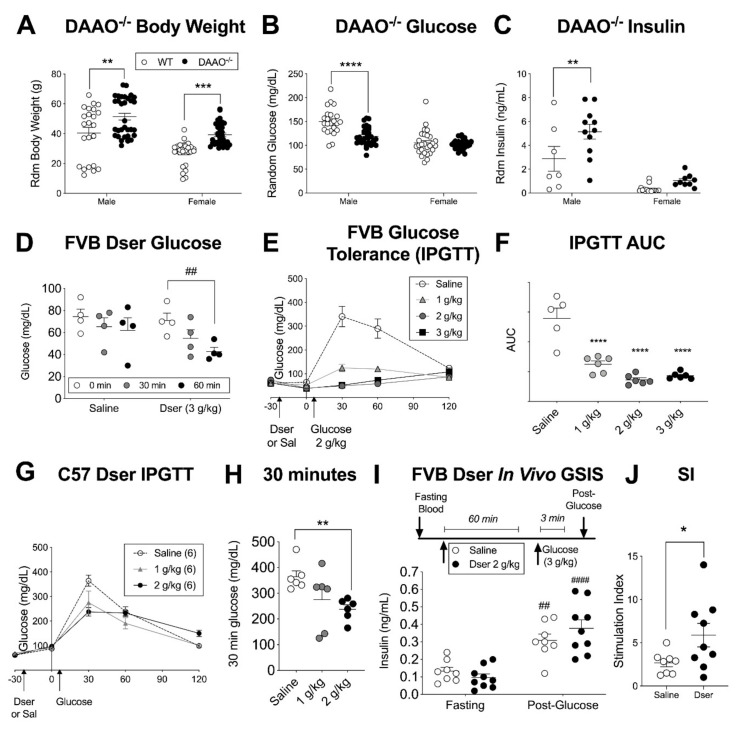
In vivo measures of glucose homeostasis in mouse models of elevated D-serine. Glucose homeostasis in a mouse with a lack-of-function mutation in the D-serine (Dser) catabolic enzyme D-amino acid oxidase (DAAO^−/−^), including (**A**) random fed body weight, (**B**) blood glucose, and (**C**) plasma insulin compared to wildtype (WT) mice on the ddY background strain. In overnight fasted wildtype mice, blood glucose responses to i.p. Dser (1–3 g/kg) were assessed in FVB strain males following (**D**) Dser alone and (**E**) when injected 30 min prior to an i.p. glucose (2 g/kg) tolerance test (IPGTT) including (**F**) area under the curve (area-under-the-curve (AUC) between 0 and 120 min post-glucose). (**G**) A similar Dser + IPGTT was conducted in C57 male mice (**H**) with an analysis of the 30-min post-glucose time point. (**I**) To evaluate in vivo insulin secretion, Dser or saline was administered 60 min prior to i.p. glucose (3 g/kg) with facial vein plasma insulin evaluated during fasting and 3 min after glucose as well as (**J**) the relative stimulation index (post-glucose/fasting). Statistical analysis of multi-endpoint data was by 2-way ANOVA with Sidak’s multiple comparison ((**A**–**C**), genotype × sex) or with repeated measures ((**D**,**I**) treatment × time). Panels F and H were analyzed by 1-way ANOVA with Dunnet’s multiple comparisons. Panel H was analyzed by 2-tailed t-test. * *p* < 0.05, ** *p* < 0.01, *** *p* < 0.001, **** *p* < 0.0001 vs. control (WT, vehicle). ## *p* < 0.01, #### *p* < 0.0001 vs. within genotype basal condition (fasting, LG).

Procedures for the in vivo assessment of glucose homeostasis have been previously described [1,26]. In brief, intraperitoneal glucose tolerance (IPGTT) and insulin tolerance testing (ITT) were tested by monitoring tail vein blood glucose before and after an i.p. bolus of glucose (2 g/kg at 0.5 g/mL, Pfizer, New York City, NY, USA) or insulin (0.75 U/kg at 0.1 U/mL, Humalog, Eli Lilly, Indianapolis, IN, USA) in 14-h or 6-h fasted mice, respectively. For in vivo GSIS, facial vein blood was collected from overnight fasted animals and 3 min after a 3 g/kg i.p. injection of glucose. For some experiments, i.p. D-serine (Sigma Aldrich, St. Louis, MO, USA, 1, 2 or 3 g/kg at 0.1, 0.2 or 0.3 g/mL in saline, respectively) or a saline control was administered in conjunction with these tests, as described in the text. Plasma was collected by facial vein into EDTA-coated tubes and assayed with the ALPCO Ultrasensitive Mouse Insulin ELISA according to kit instructions. Figure 1 experiments on DAAO^−/−^ mice included blood glucose and body weight data collected from the entire colony of adjacently reared mutant and ddY WT mice between 3 and 14 months of age on 2 occasions approximately 1 year apart. The remaining tests were assessed in the colony at a single timepoint. Plasma insulin was determined by facial vein blood samples in randomly fed animals or after a 4–6 h fast in DAAO^−/−^ mice and controls. The detection of D-serine by capillary electrophoresis in the facial vein serum of random fed mice from this colony was thoroughly described in a previous publication [27].

### 2.3. Mouse Islet Experiments

As previously described [1,26], pancreatic islets were isolated via ductal inflation with 0.75 mg/mL collagenase P (Roche 11213865001) and handpicked clean into RPMI media for an overnight rest in a humidified incubator at 37 °C, 5% CO_2_ before use. For in vitro GSIS, islets were pre-incubated for 2 h in 2 mM (low) glucose in a Krebs buffer, then 10 islet aliquots were picked into cell culture inserts in a 24-well plate (2–3 wells per condition per *N*). Islets were incubated sequentially in low glucose (LG) and then high glucose (22 mM, HG) for 30 min each. For some experiments, a combination of NMDA (Sigma Aldrich, M3262) and/or D-serine (Sigma Aldrich, S4250), dissolved in sterile water to 10, 100, or 1000 mM stocks, was diluted into LG/HG solutions 1000-fold to their final concentrations. GSIS insulin secretion is presented normalized to the total insulin content of the islets collected at the end of the experiment. Islet insulin content was calculated as the sum of the total insulin released and the final islet insulin, normalized to the DNA of the collected islets in each well (Quant-iT Pico-Green dsDNA Assay Kit, Molecular Probes, Eugene, OR, USA). With the exception of the high fat diet (HFD) experiment, all GSIS data was derived from at least 2 independent experiments.

### 2.4. Gene Expression

For the analysis of background strain gene expression, TPM (transcripts per kilobase million) values were extracted from the RNAseq data files uploaded to the Genome Expression Omnibus data (GSE123893) as a supplementary component of a recent publication by Zhang et al. [28]. Only those files corresponding to hypothalamic tissue from water-drinking C57 mice (GSM3515220-23) and water-drinking FVB mice (GSM3515236-39) were used. Values were averaged across presumed replicates (L6–8) with two replicates excluded (CW4_L6, FW8_L7) for having predominantly 0 coverage of the candidate gene set, which included Srr, Dao, Grin1, Grin2a, Grin2b, Grin2c, Grin2d, Grin3a, Grin3b. qPCR on isolated islets was performed as previously described [1] but values are presented as normalized to Grin1, to represent subunit proportion relative to potential overall channel number. In addition to the qPCR primers listed in the referenced publication for β-actin, Grin1, Grin2a, and Grin2b, the following sequences were used for Grin2c (AGACCAATACCCACCCTTCC, GCCATGTTGTCAATGTCCAG), Grin2d (CGATGGCGTCTGGAATGG, CTGGCAAGAAAGATGACCGC), Grin3a (GGTCCACCCGGCTCCCGAAAG, TTGGAGTTATTCTCCGTAAGGAGGAGGAA), taken from [29]. Normalization of dCT values to Grin1 was on a per mouse basis.

### 2.5. Electrophysiology

As previously described [26], dispersed islets (0.25% trypsin) were seeded onto coverslips or intact islets were held in place by suction to a polished glass pipette. Individual β-cells were targeted based on size and shape and confirmed based on excitatory responses to stimulatory (12 mM) glucose. Amphotericin-B was applied following giga-seal formation to obtain a perforated patch current-clamp for recording membrane potential, series resistance and cell capacitance. The perforated patch intracellular solution contained (in mM): 76 K_2_SO_4_, 10 NaCl, 10 KCl, 1 Mg_2_Cl_2_, 5 HEPES, 0.5 EGTA and 0.25 mg/mL of amphotericin B, pH7.3 with KOH. Various drugs (3 or 12 mM glucose, 100 μM NMDA, 100 μM D-serine, 50 μM 7-DCKA) were gravity-perfused into the recording chamber with the extracellular solution (in mM: 115 NaCl, 3 CaCl_2_, 5 KCl, 2 MgCl_2_, 10 HEPES, with mannitol for osmolarity, pH 7.4 with NaOH) through a custom chamber at a rate of 1.5–2 mL/min. Patch electrodes were pulled from borosilicate glass using a Sutter P-97 puller to obtain a tip resistance of 3–8 MΩ. Whole-cell recordings were made using an Axon Instruments Multiclamp 700B, digitized with a Digidata 1320A, and analyzed in P-clamp and Origin software.

### 2.6. Immunostaining

After harvesting and fixing whole pancreata in 3.7% formaldehyde, embedded tissue was cut into 5 μM sections for staining, as described [1,26]. Sections were stained with a guinea pig primary antibody against insulin (1:100, Abcam Ab7842) and a secondary antibody conjugated to Cy3 (Jackson Immunoresearch, 706-165-148) then coverslipped with DAPI mounting medium and imaged on a Nikon Eclipse Ni-E microscope. β-cell mass was determined as previously reported [1,25] to be insulin-positive area/pancreas area, averaged over 5 sections taken 200 μM apart, multiplied by pancreas weight. DAB immunochemical staining for GluN3a (Santa Cruz Biotechnology, sc-98986), shown in the supplement, was produced according to our previously described protocol [1].

### 2.7. Statistical Analysis

Single endpoint data was evaluated by a 2-tailed *t*-test, independent unless stated to be paired, except in the case of individually normalized electrophysiology data, which was assessed by a 1-sample *t*-test. Multi-endpoint (e.g., IPGTT) or multi-factor (e.g., sex and genotype) data was analyzed by 1 and 2-way analysis of variance (ANOVA) with repeated measures and specific post-hoc analyses when indicated, as specified in the figure legend. In vivo data was analyzed with sex as a segregate factor, but significant sex differences, independent of genotype, are not listed for clarity. In vitro islet data was collected from both sexes but combined indiscriminately during analysis. In most cases, data is presented as dot plots to indicate group numbers and scatter. Where line graphs or bar and whisker (min-to-max) plots were used for visual clarity, group numbers are given in the legend and an effort was made to present a secondary analysis (e.g., area-under-the-curve, AUC) to represent scatter. Graphs show average data with error bars indicating standard error of the mean. Statistical analyses, including AUC, were calculated using GraphPad Prism v7.0 with significance set at *p* < 0.05.

### 2.8. Study Approval

All animal procedures were approved by the Institutional Animal Care and Use Committee at the University of Minnesota (protocol #1806-36072A).

## 3. Results

### 3.1. Acute Systemic D-serine Lowers Blood Glucose in Multiple Mouse Strains

Mice with a constitutive loss of the D-serine catabolic enzyme D-amino acid oxidase (DAAO) have a life-long overabundance of systemic D-serine [30,31], including a more than two-fold increase in circulating levels [32], which we re-iterated for serum D-serine in our own colony (Supplemental Figure S1). We further report that these mice exhibit a colony average higher body weight, lower blood glucose, and elevated plasma insulin levels (Figure 1A–C) but there was a significant sex interaction effect with the relative hypoglycemia and hyperinsulinemia more apparent in males than females. This was surprising based on previous studies showing improved glucose tolerance and insulin secretion in mice with a loss of D-serine synthetic capacity [1] or D-serine-targeted NMDARs in the pancreas [6]. We therefore sought to more directly examine the impacts of systemic D-serine on glucose homeostasis following acute i.p. injection. Preliminary experiments in randomly fed male and female mice suggested that D-serine (3 g/kg) may lower blood glucose within a 2-h timeframe in ddY mice (DAAO^−/−^ background strain) and the more common FVB strain (Supplemental Figure S2A,B) but was not as effective in C57 mice (Srr KO background strain, Supplemental Figure S2C). This was confirmed in a higher-powered run of overnight fasted FVB male mice, which showed a significant 20% decrease in blood glucose one hour after D-serine administration, compared to the relatively stable values in saline-injected controls (Figure 1D). Furthermore, when D-serine was administered 30 min prior to i.p. glucose (2 g/kg), it dose-dependently improved i.p. glucose tolerance (IPGTT) (Figure 1E,F). We repeated this experiment in fasted C57 male mice (Figure 1G) and although glucose tolerance was improved at 30 min in the D-serine (2 g/kg) group (Figure 1H), the magnitude of effect was smaller than in the FVB mice, similar to our preliminary findings. We then probed whether this glucose-lowering effect was related to changes in insulin secretion by pre-injecting D-serine 1-h prior to a high glucose bolus (3 g/kg i.p.) and assessing plasma insulin in both pre-treatment fasting and post-glucose samples (Figure 1I). Indeed, we found a significant increase in the ratio of these two values (the stimulation index, SI) in D-serine vs. saline-treated FVB male mice (Figure 1J) indicating a potentiation of in vivo GSIS in response to systemic D-serine.

### 3.2. D-Serine with NMDA Potentiates Glucose-Stimulated Insulin Secretion and β-Cell Excitation

**Figure 2:**
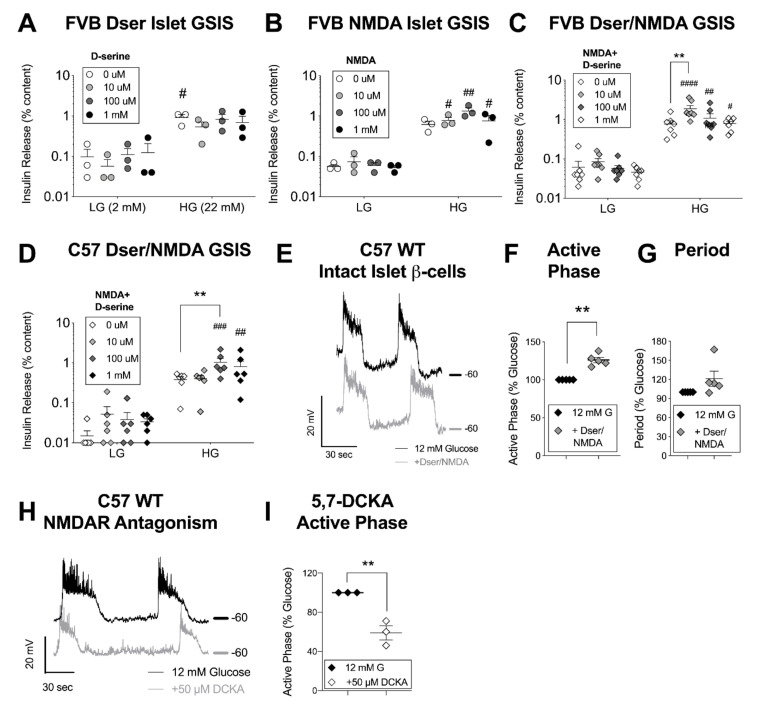
In vitro insulin and β-cell membrane responses to N-methyl D-aspartate receptor (NMDAR) activation with D-serine co-agonism. Isolated islets from FVB mice were assessed for insulin secretion (as % islet insulin content) in response to low glucose (LG, 2 mM), high glucose (HG, 22 mM), and a concentration range of (**A**) D-serine, (**B**) NMDA, or (**C**) a range of D-serine with 100 μM NMDA. (**D**) D-serine + 100 μM NMDA glucose-stimulated insulin secretion (GSIS) experiments were repeated with C57 mouse strain-derived islets. A perforated patch clamp technique was used to record β-cell membrane potentials from intact C57 WT islets. (**E**) Representative traces show oscillations from the same cell ~10 min after incubation in 12 mM glucose (black) and ~ 1 min after exposure to 100 μM each of Dser/NMDA (grey); −60 mV level is shown for each trace. (**F**) Active phase duration (width of depolarized plateau) and (**G**) period between the start of each oscillation was analyzed just before and after treatment. (**H**) Representative traces from a similar experiment on islet β-cells before and after treatment with the NMDAR (GluN1) D-serine binding site antagonist 5,7-dichlorokynurenic acid (5,7-DCKA) and (**I**) a quantitative analysis of active phase duration. Data in circles was derived from male animals and data in diamonds were mixed sex. Islet GSIS was statistically analyzed by 2-way ANOVA (glucose × treatment) with repeated measures. Oscillation characteristics (normalized to the glucose baseline on a per cell basis) were assessed by 1-sample *t*-test. # *p* < 0.05, ## *p* < 0.01, ###*p* <0.001, #### *p* < 0.0001 vs. similar condition LG level. ** *p* < 0.01 vs. control, as indicated. GSIS data is presented on a y-axis logarithmic scale to facilitate evaluation of LG values.

Chronic D-serine and/or NMDAR activity in the CNS has been linked to the indirect regulation of insulin secretion and blood glucose in previous studies [5,33,34]. To isolate these effects from any direct impact of D-serine on β-cell insulin secretion, we isolated primary pancreatic islets from the FVB strain of mice and subjected them to an in vitro GSIS with varying concentrations of D-serine (0–1000 μM) supplementing all incubation solutions. D-serine alone had no significant acute impact on islet insulin secretion (Figure 2A) nor did a dose-range of the highly specific NMDAR channel agonist NMDA (Figure 2B), which re-iterates previously published results [5,6]. However, the combination of 100 μM NMDA and 10 μM D-serine strongly potentiated stimulated insulin secretion without effecting basal release (Figure 2C). We conducted this latter experiment in islets from both male and female mice, but after observing no sex difference in the pattern of response, combined the results for a higher-powered analysis. Repeating the same experiment in C57 male and female islets, 10 μM D-serine + 100 μM NMDA was insufficient but 100 μM D-serine (+NMDA) potentiated GSIS (Figure 2D), continuing the strain-based pattern of reduced D-serine potency in C57 mice noted in the in vivo results.

To probe this difference a little further, we accessed recently published RNAseq gene expression data (GSE123893) from C57 and FVB hypothalamic tissue [28], examining a pre-determined set of D-serine related genes. This analysis revealed decreased FVB expression of serine racemase (Srr) and the NMDAR “inhibitory” subunit [35], Grin3a (Supplemental Figure S3A,B). We found a similar difference in islet Grin3a expression by qPCR, measuring transcripts in half of the C57 islet samples but none in the FVB islets, despite an equivalent RNA load and full detection of all other Grin1 and Grin2-type subunits (Supplemental Figure S3C). Using DAB staining, we also confirmed islet-enriched protein expression of GluN3a in C57 pancreatic slices (Supplemental Figure S3D). GluN3a subunits are known to decrease NMDAR channel conductance and lower open probability [36], which may contribute to a decrease in C57 D-serine sensitivity or efficacy.

Nevertheless, as the most commonly used background strain, including for our own transgenic models, we continued to probe β-cell in vitro responses in C57 islets using the concentrations of D-serine and NMDA that were found to be experimentally effective for this strain (i.e., 100 μM each, in combination). It is worth noting here that although islet D-serine levels have not been directly measured in islets, we approximate basal pancreatic D-serine concentration at 40–117 μM, based on whole pancreas tissue content [37] and estimated pancreatic weight and volume calculation [38] for a 20 g mouse, which is well within the range of CNS concentrations of 10–400 μM D-serine [39]. So while 100 μM D-serine/100 μM NMDA has been commonly used in electrophysiology to maximize detection sensitivity through receptor saturation (e.g. in [40]), it is not inconceivable that the GSIS-activating D-serine concentrations, especially for FVB islets, are also within a range of biological relevance.

There are many mechanisms that can change the GSIS stimulus-secretion coupling relationship, but the immediate effect of D-serine, if acting as an activating co-agonist for β-cell NMDARs, should be a change in the electrical properties of the membrane during a depolarizing stimulus (i.e. glucose) sufficient to expel any charge-based channel block. To specifically test for this, we used a perforated patch-clamp approach and targeted large-diameter cells on the surface of intact islets. Cells exhibiting excitatory oscillations during a 10-min incubation in 12 mM glucose (i.e. β-cells) were subsequently bathed in D-serine/NMDA. After ~1 min of acclimation, oscillation characteristics were compared to those recorded just before the solution change (Figure 2E). We observed a significant increase in glucose-stimulated active phase duration after D-serine/NMDA (Figure 2F), while a trend towards prolonged period did not reach statistical significance (*p* = 0.15) (Figure 2G). We saw a similar D-serine/NMDA-induced increase in excitatory spike activity in recordings made from single β-cells derived from trypsin-dispersed islets (Supplemental Figure S4). Furthermore, pharmacological antagonism of the NMDAR D-serine binding site, using 5,7-dichlorokynurenic acid (5,7-DCKA) on intact islet β-cells and experimental timing as described, depressed active phase duration (Figure 2H,I), suggesting some degree of islet-level native NMDAR activity during stimulatory glucose. We confirmed the specificity of drug action through the GluN1 co-agonist binding site by adding back in D-serine, which reversed the suppression of active phase duration in two cases from 60% and 51% of baseline with 5,7-DCKA alone to 96% and 97% of baseline with 5,7-DCKA + 200 μM D-serine in the same cell. Collectively, these results support the idea that NMDAR activation through acute D-serine co-agonism enhances β-cell excitation and insulin secretion.

### 3.3. β-Cell Specific Loss of NMDAR Co-Agonist Binding Protein Negates In Vitro D-Serine/NMDA Effects

**Figure 3:**
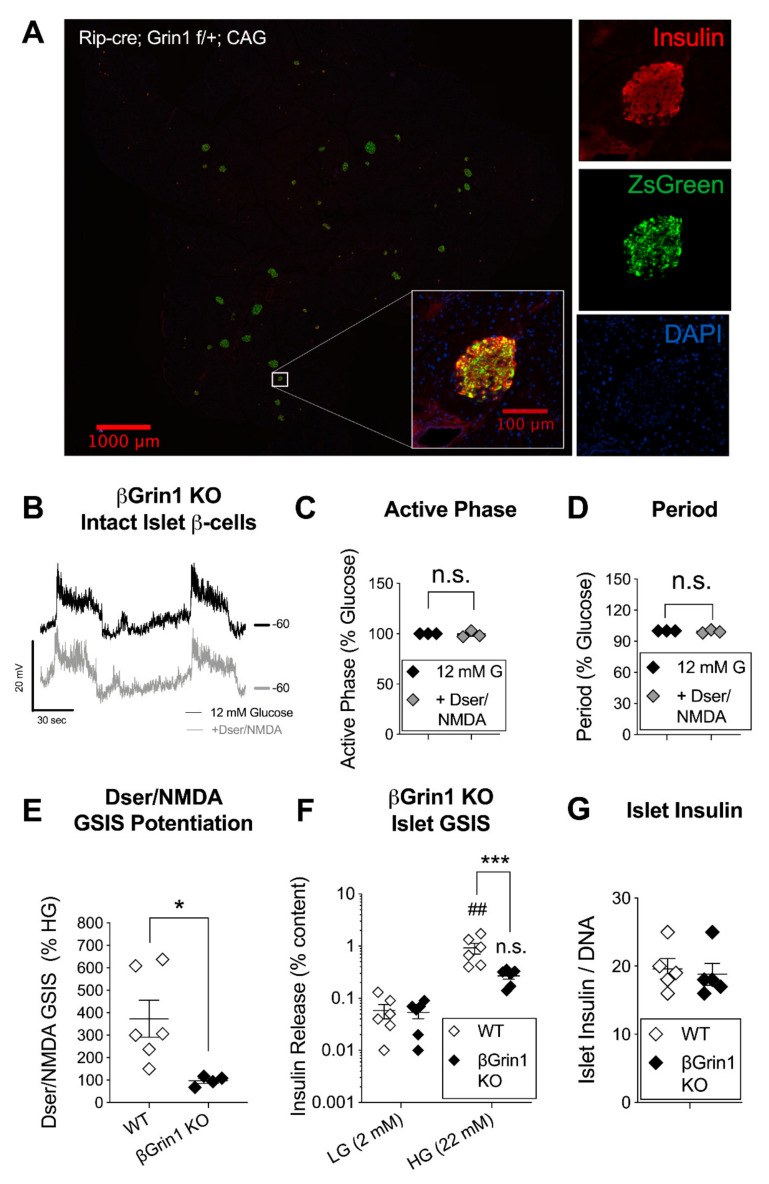
Dser/NMDA membrane and functional responses in a mouse model with β-cell specific GluN1 loss. Ins2-driven Cre-Lox recombination of the Grin1 gene (Rip-cre; Grin1 f/f, βGrin1 KO) was used to achieve β-cell specific loss of D-serine-binding GluN1 protein (and thus all NMDARs) in C57 mice. (**A**) Immunofluorescent staining of a fixed pancreatic slice (10x, left) and a single islet (20x, right) from a Rip-cre; Grin1 f/+ mouse bred with a CAG-ZsGreen reporter gene showing colocalization of Cre-driven green fluorescence and β-cell insulin signals. (**B**) Representative recordings of glucose-stimulated (12 mM) membrane voltage oscillations in β-cells from intact βGrin1 KO islets immediately before (black) and ~1 min after D-serine/NMDA (100 μM each, grey) with a comparative quantification of (**C**) active phase duration and (**D**) period. (**E**) The relative effect of the same concentration of D-serine/NMDA on GSIS (22 mM glucose) in WT (Cre-negative; Grin1 f/f or Grin1 f/+) and βGrin1 KO islets and the effect of genotype on islet (**F**) insulin secretion and (**G**) insulin content in an independent GSIS experiment. Data in panels C, D were analyzed by 1-sample *t*-test and in E, G by independent 2-tailed *t*-test. Data in panel F were analyzed by repeated measures 2-way ANOVA. Not significant (n.s.) *p* > 0.05, ## *p* < 0.01 vs. w/in genotype LG or as indicated. * *p* < 0.05, *** *p* < 0.001 vs. WT.

The timing and mechanistic specificity of these experiments are crucial to their interpretation as recent studies have also shown that NMDAR activity can influence β-cell excitability indirectly through the expression or function of K_ATP_ channels [16]. So to be certain that the stimulatory effects of acute D-serine were dependent on its NMDAR co-agonism, we bred C57 mice with a β-cell specific deletion of the Grin1 gene, which encodes for the GluN1 NMDAR subunit that contains both the co-agonist (D-serine) binding site and is required for functional oligomerization of the receptor channel [41]. Excision of floxed Grin1 DNA was driven by the insulin 2 gene promoter (Rip-cre [42]), resulting in a robust and β-cell (insulin+) specific Cre expression in pancreatic tissue, with no apparent change in the number or visual appearance of islets and islet cells (Figure 3A). However, βGrin1 KO membranes were insensitive to the application of D-serine/NMDA with no changes in the active phase or period of intact islet β-cell oscillations (Figure 3B–D) or in the spike rate of dispersed islet β-cells (Supplemental Figure S5). Qualitatively, we noted an apparent decrease in the stability of βGrin1 KO membrane polarization relative to WT recordings, but due to inherent biological and cellular heterogeneity (for example, characterized in [16]), we did not attempt a quantitative or statistical comparison at this group size. Nevertheless, we were confident that D-serine/NMDA potentiation of islet insulin secretion was lost in βGrin1 KO islets, with an average D-serine/NMDA GSIS at 96% of the glucose-only response (*N* = 4 mice, 2–3 10-islet replicates per *N*) vs. ~373% in WT (*N* = 6 mice) (Figure 3E). In addition, absolute GSIS was significantly depressed in βGrin1 KOs relative to parallel-run WT controls (Figure 3F). This difference in secretory capacity was evidently functional as we observed no genotype-based change in islet insulin content (Figure 3G). Thus, the in vitro data from this model demonstrates that the β-cell excitation and insulin-stimulating effects of acute D-serine depend on the capacity for β-cell NMDAR activation.

### 3.4. Minimal In Vivo Phenotype but Age-Related Reversal of βGrin1 KO Secretory Phenotype

**Figure 4:**
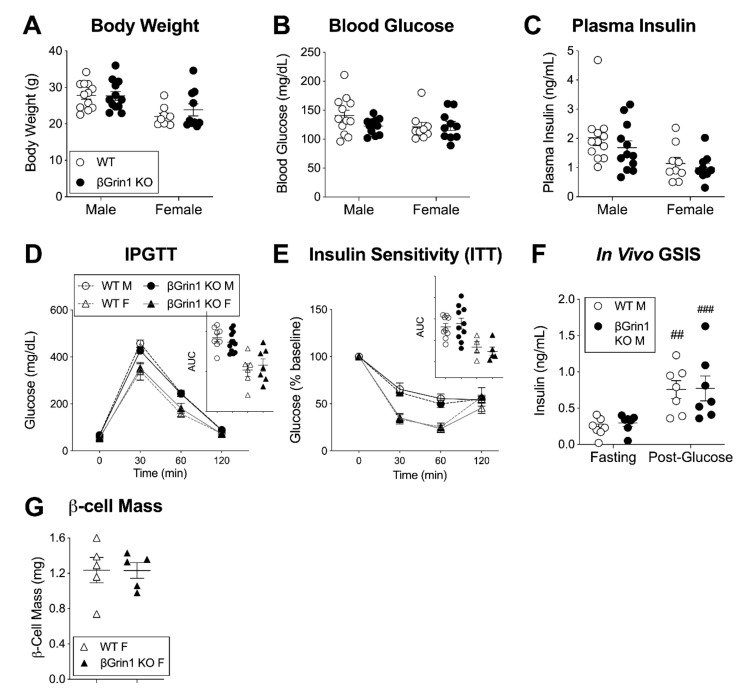
In vivo metabolic phenotype of young adult βGrin1 KO mice. WT and βGrin1 KO male and female mice were separately evaluated for random fed (**A**) body weight, (**B**) blood glucose, and (**C**) plasma insulin. (**D**) Glucose tolerance was assessed in male (circles) and female (triangles) overnight fasted mice and (**E**) insulin sensitivity in 6-h fasted mice following 0.75 U/kg ip insulin. (**F**) An in vivo GSIS was also conducted in male mice and (**G**) β-cell mass was determined by immunofluorescent insulin staining of fixed pancreatic sections from female mice. Statistically, single endpoint data (**A**–**C**,**G**), AUC) was assessed by independent 2-tailed *t*-test and multi-endpoint data (**D**–**F**) by repeated measures 2-way ANOVA for each sex. ## *p* < 0.01, ### *p* < 0.001 vs. w/in genotype fasting level. ITT = Insulin tolerance test. Cre-negative (Grin1 f/f or Grin1 f/+) mice were used as WT controls.

Both specific antagonism of the GluN1 D-serine binding site and its tissue-specific deletion led to a depression of glucose-stimulated β-cell excitatory characteristics, which suggests that endogenous NMDAR activity promotes acute β-cell function. However, this stands in contrast to recent hypotheses characterizing NMDARs with a net inhibitory role on β-cell function and glucose tolerance [5,6,16] including a model of Ins1-Cre driven β-cell GluN1 deletion that showed impaired islet GSIS [6]. Given our dissimilar in vitro results, we therefore sought to investigate glucose homeostasis more generally in our βGrin1 KO model using male and female mice in the 2–4-month age range that corresponded to diminished βGrin1 KO islet GSIS. Surprisingly, we didn’t see any difference between the in vivo phenotype of WT and βGrin1 KO mice, including random fed body weight, blood glucose, and plasma insulin (Figure 4A–C). While the mice did show characteristic sex-differences in i.p. glucose and insulin tolerance testing (Figure 4D,E), genotype accounted for less than 0.25% of total data variation within any single-sex test analysis. A male-only in vivo GSIS also showed similar fasting and glucose-stimulated systemic insulin levels for both genotype groups (Figure 4F). We considered whether an increase in β-cell mass might be compensating for secretory dysfunction but saw no genotype-related differences among female pancreata on this measure (Figure 4G).

We continued to probe for a metabolic phenotype in βGrin1 KO mice by increasing insulin secretory demand through up to 14 weeks of high fat diet (HFD, 60% kcal fat, 3 months starting age). Among males, WT and βGrin1 KO mice gained weight comparably (Supplemental Figure S6A) and demonstrated equivalent blood glucose at ~4-week intervals throughout (Supplemental Figure S6B). Glucose tolerance, insulin sensitivity, and in vivo GSIS (Supplemental Figure S6C–E), tested at 8, 6, and 14 weeks HFD respectively, were also not different between genotypes. By contrast, female βGrin1 KO mice were somewhat resistant to diet-induced obesity with lower average body weight at 10 and 13 weeks of HFD compared to WT controls (Supplemental Figure S7A) and the absence of mild hyperglycemia, observed in WT females between weeks 2 and 13 (Supplemental Figure S7B). However, glucose tolerance, insulin sensitivity and plasma insulin, at 7, 5, and 13 weeks HFD respectively, were not different (Supplemental Figure S7C–E), nor were islet GSIS (Supplemental Figure S7F) and β-cell mass (Supplemental Figure S7G) after 13 weeks.

**Figure 5:**
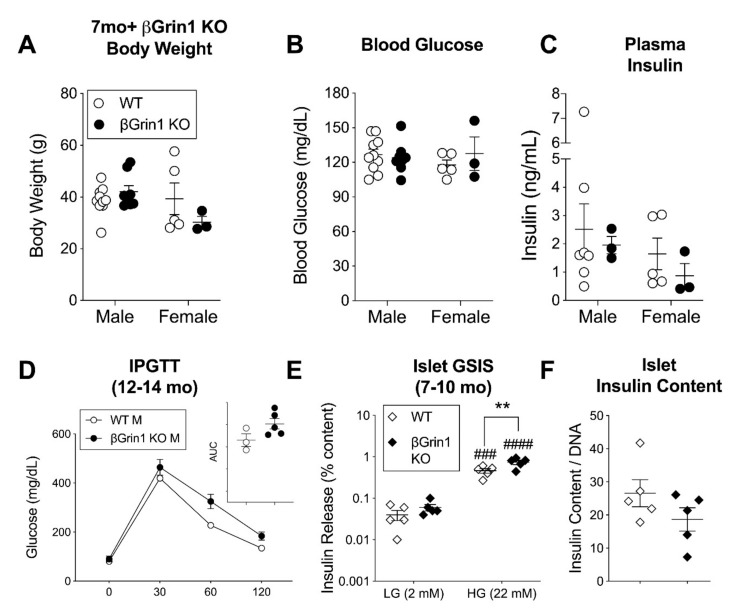
In vivo phenotype of older adult βGrin1 KO mice. Male and female βGrin1 KO and WT mice, aged 7–14 months, were assessed for measures of glucose homeostasis including (**A**) random fed body weight, (**B**) blood glucose, and (**C**) plasma insulin; (**D**) 12–14-month-old males were also given an IPGTT with AUC shown in the inset. Islet data derived from 7–10-month-old male and female mice from each genotype was combined (diamonds) to show (**E**) islet insulin secretion by GSIS and (**F**) total islet insulin content. Statistical analysis was as described for similar data in previous figures, with independent Student *t*-tests and repeated measures 2-way ANOVA for within sex single and multi-endpoint data, respectively. ### *p* < 0.001, #### *p* < 0.0001 vs. within group LG. ** *p* < 0.01 vs. WT, as indicated. Figure legend defines all subsequent panels until re-defined. Cre-negative (*Grin1* f/f or *Grin1* f/+) mice were used as WT controls.

As another form of metabolic stress, we tried testing older animals (aged 7–14 months), but again body weight, blood glucose, and plasma insulin were equivalent between WT and βGrin1 KO mice of both sexes (Figure 5A–C). An IPGTT in 12–14-month-old males trended higher in the NMDAR-loss model without reaching significance (*p* = 0.10 for AUC) (Figure 5D). Remarkably, in vitro GSIS was slightly higher in islets from older βGrin1 KO mice compared to age-matched WT controls (mixed sex) (Figure 5E), again without a change in total islet insulin content (Figure 5F). This age-related reversal in the effect of β-cell NMDAR loss on in vitro insulin secretion offers an alternative explanation for the lack of genotype effect on GSIS in the HFD experiment, which by nature of its design was derived from 7–8-month old animals. Furthermore, this hypersecretory phenotype re-capitulates previously published findings from Ins1-Cre; Grin1 KO islets [6], potentially reconciling the two datasets and offering a novel explanation for contradictory observations in the literature on anti-diabetic [6] and pro-diabetic [43] effects of the NMDAR antagonist dextromethorphan, with the latter report specifically in children.

### 3.5. Sex-Specific Glucose Intolerance in a Mouse Model with Pancreatic Deletion of GluN1

Loss of β-cell GluN1 had a significant, if context-dependent, effect on in vitro insulin secretion, but the lack of a concomitant in vivo phenotype under any condition leaves an open question as to the relevance of this model to understanding either the endogenous role of β-cell NMDARs or D-serine’s modulation of their activity. One possibility is that this model fails to capture the influence of non-β-cell islet cells, which have recently been shown to express NMDARs [16]. To overcome this limitation, we crossed Pdx1-Cre [42] into our Grin1 floxed line to achieve a pancreas-wide deletion of GluN1. We re-iterated our previous description of the high pancreatic expression efficacy of this Cre using the CAG-ZsGreen reporter gene [44] which reveals a strong endogenous pancreatic fluorescence even under normal light conditions (Supplemental Figure S8). Marquard et al. characterized a similar mouse model using a variant Pdx1-Cre driver [45] and comparing Pdx1-Cre; Grin1 f/f (pGrin1 KO) male mice against Pdx1-Cre; Grin1 f/+ (pGrin1 Het) male controls [6]. Therefore, we were careful to include heterozygous mice in our own experiment, along with pGrin1 KO and Cre-; Grin1 floxed controls (WT). We were also mindful of the sex differences repeatedly observed in various models of this system (Srr KO, DAO mutant, HFD βGrin1 KO) and ran fully powered independent assessments of both male and female mice for all genotype groups in a 3–5-month age range.

**Figure 6:**
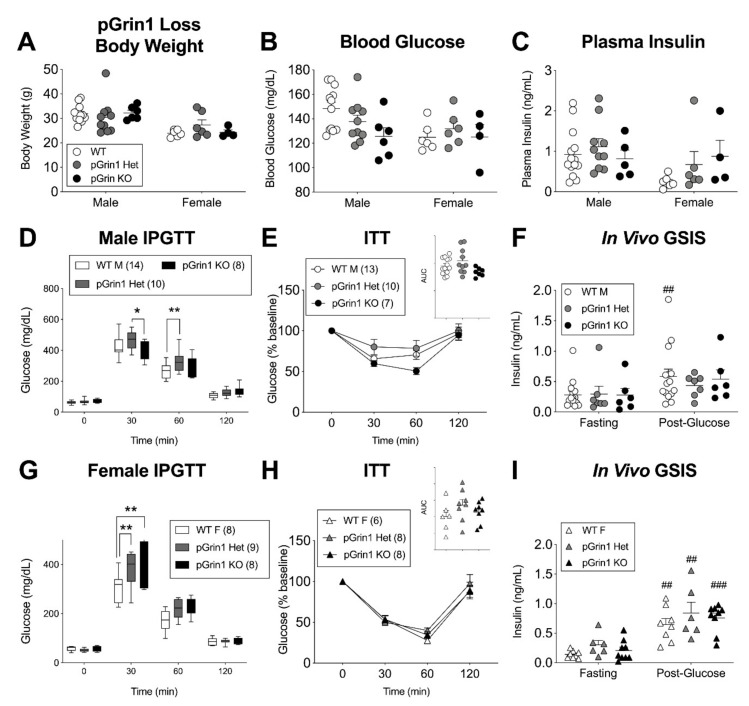
Glucose intolerance in young adult pancreatic Grin1 loss mice. A Pdx1-Cre driver was used to partially (pGrin1 Het, grey) or fully (pGrin1 KO, black) knockdown GluN1 protein in all pancreatic tissue; 3–5-month-old mice were phenotyped against Grin1 f/f or f/+ control mice (WT). Male and female random fed mice were evaluated for (**A**) body weight, (**B**) blood glucose, and (**C**) plasma insulin. As previously described, male mice were assessed for (**D**) glucose tolerance, (**E**) insulin sensitivity, and (**F**) in vivo GSIS. Female mice were also tested for (**G**) glucose tolerance, (**H**) insulin sensitivity, and (**I**) in vivo GSIS. Data in all panels were evaluated by repeated measures 2-way ANOVA with Tukey’s (**D**,**G**) or Sidak’s (**F**,**I**) multiple comparisons. ## *p* < 0.01, ### *p* < 0.001 vs. fasting. *, ** *p* < 0.05, 0.01, vs. WT control.

We saw no differences between WT, pGrin1 Het, and pGrin1 KO random fed body weight, blood glucose, or plasma insulin levels for either sex (Figure 6A–C). In males, there was a small but significant improvement in pGrin1 KO IPGTT glucose tolerance at 30 min relative to pGrin1 Het, which reflects the previously published model results [6], but we also saw glucose intolerance at 60 min between the WT and pGrin1 Het males in the same test (Figure 6D). During insulin tolerance testing, genotype neared significance as a factor (*p* = 0.07 by 2-way ANOVA) with pGrin1 KO trending towards increased sensitivity (Figure 6E). Similarly, the change in insulin release between post-glucose and fasting glucose was somewhat more robust in the WT mice vs. the GluN1 loss models, but ultimately neither insulin sensitivity nor in vivo GSIS were demonstrably different between male groups (Figure 6F). In the female mice, we found more consistent evidence of glucose intolerance for both pGrin1 Het and pGrin1 KO mice compared to WT controls at the 30-min IPGTT timepoint (Figure 6G), but insulin sensitivity and in vivo GSIS were unaffected (Figure 6H). In sum, pancreas-wide deletion of GluN1 in young adult mice resulted in several indicators of glucose intolerance, and some trends in male mice consistent with insulin hyposecretion, although the potential influence of glucose-regulating NMDAR activity in the hypothalamus [46], where Cre expression has been demonstrated for both our promoter constructs [47,48], cannot be ruled out.

## 4. Discussion

**Figure 7:**
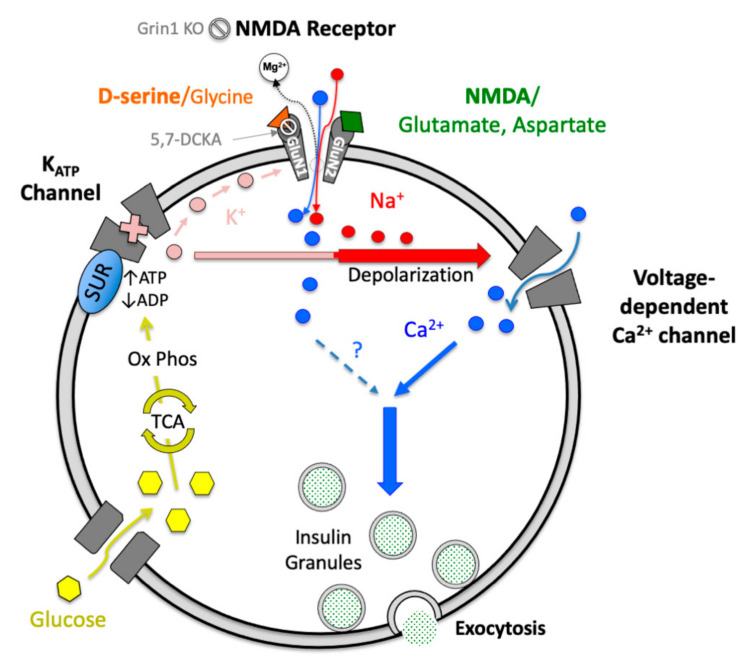
A hypothetical excitatory model of β-cell NMDAR activity. In canonical β-cell stimulus-secretion coupling, glucose oxidation stimulates the consumption of ADP and production of ATP, which together inhibit K^+^-effluxing K_ATP_ channels, leading to local depolarization and the activation of voltage-sensitive L-type Ca^2+^ channels, culminating in the Ca^2+^-triggered exocytosis of insulin granules. When D-serine and NMDA (exogenous) or glutamate (endogenous) are present in the extracellular space during sufficient intracellular depolarization to eject the NMDAR Mg^2+^ channel block, positive ionic flux through the channel enhances the signal for exocytosis, directly or through a stronger activation/recruitment of Ca^2+^ channels, resulting in potentiation of insulin secretion. Mechanism of NMDAR inhibition is indicated for 5,7-DCKA and Grin1 KO. TCA = tricarboxylic acid cycle. Ox Phos = Oxidative Phosphorylation.

The current investigation showed that acute systemic D-serine lowered blood glucose and increased in vivo insulin secretion in mice. D-serine also potentiated islet insulin secretion in vitro, but only when exogenous conditions were provided to ensure the capacity for NMDAR activation—high glucose to stimulate local membrane depolarization, the simultaneous presence of both agonist (NMDA) and a sufficient concentration of co-agonist (D-serine) and β-cell expression of the co-agonist binding GluN1 receptor subunit. D-serine-mediated NMDAR activation resulted in an acute enhancement of β-cell membrane excitation characteristics, in a pattern similar to other endogenous GSIS-enhancing agents [49]. These data are consistent with a classical model of NMDARs in the CNS in which the receptor-channel’s facilitation of positive intracellular ionic flux enhances membrane excitability and strengthens the relationship between synaptic signaling and neurotransmitter exocytosis. Such a system is easily envisioned within the canonical β-cell stimulus-secretion pathway, falling between K_ATP_ channel closure and the activation of L-type Ca^2+^ channels, possibly also contributing to net [Ca^2+^]_i_, to enhance glucose-stimulated insulin secretion (Figure 7).

A D-serine excitatory β-cell model, while consistent with our data, raises questions about how to interpret a recent report that showed chronic D-serine drinking (0.5–1%) leads to hyperglycemia, reduced weight gain, and hypoinsulinemia [5], an outcome we have also seen in our own unpublished observations of a similar model (~1% w/v D-serine in water). It is important to note, however, that Suwandhi et al. also showed robust hyperinsulinemia when a lower dose of in vivo D-serine (0.1%) was used in the same experimental design. Similarly, we found D-serine concentration to have a critical effect on outcome, with concentrations above or below a crucial range failing to potentiate islet GSIS. This could reflect D-serine’s stimulation of secondary changes in membrane plasticity, as repeated high dose D-serine has been shown to trigger NMDAR internalization [11], as well as NMDAR activation itself potentially leading to increased trafficking of K_ATP_ channels to the cell surface [16]. Thus, acute and low-dose D-serine may primarily activate existing NMDARs, leading to depolarization, while longer, higher-dose exposures stimulate re-modeling that reduces overall β-cell excitability and function.

Another possibility derives from potentially damaging side effects of high D-serine activity. This includes excitotoxicity from NMDAR overactivation [50], which has already been implicated in hyperglycemic β-cell dysfunction and death [17], and the production of H_2_O_2_ during DAAO-mediated D-serine catabolism, resulting in oxidative stresses [51] to which β-cells are particularly vulnerable [52]. Importantly, we do not believe that toxic cell death was a major contributor to the acute in vitro outcomes in this study, based on preliminary assessments in human islets that showed 100% viability after a 60-min incubation in the maximally stimulatory 100 μM D-serine/100 μM NMDA/28 mM glucose (*N* = 2 replicates, data not shown). However, the balance point between the pro-secretory NMDAR-activating potential of D-serine and a cell’s proclivity towards homeostatic membrane plasticity or susceptibility to toxic stress could contribute to differences in response to acute and chronic D-serine, low and high dose D-serine, and strain-based shifts in the bounds of this efficacy window. In vivo, CNS effects must also be considered as high concentration or repeated dosing of D-serine can accumulate beyond the blood-brain barrier where hypothalamic NMDAR activity, especially in the paraventricular nucleus, has been linked to hepatic gluconeogenesis [34] and to adrenergic modulation of insulin secretion [5].

A close attention to context, and the potential for plasticity and off-target effects, may also be needed to interpret and resolve the apparently divergent conclusions of past investigations into the endogenous role of β-cell NMDARs. A trio of studies in the mid-1990s used mouse insulinoma cells and primary rat islets to show excitatory and/or pro-secretory effects of NMDA + glycine [12,14,53],but more recent studies have promoted an inhibitory model of β-cell NMDARs [6,16], where an initial activation-induced depolarizing current acts as a trigger for other cellular mechanisms (e.g. K_ATP_ trafficking/activation) that have a net hyperpolarizing effect on the cell. Much of this latter hypothesis is derived from the observation that NMDAR antagonists, primarily MK-801 and dextromethorphan (DXM), potentiate insulin secretion [6], a finding which has been independently re-iterated [1,5,54], although not universally so [15,17]. However, the specific mechanisms through which these drugs act are unclear. For example, DXM was recently shown to directly antagonize K_ATP_ channels, and to a lesser degree L-type Ca^2+^ channels, leading to K_ATP_-dependent GSIS potentiation but also to GSIS inhibition when used at a higher concentration [54]. Similarly, MK-801 is known to have non-NMDAR targets with functional β-cell expression including nicotinic acetylcholine receptors [55,56] and the 5-HT transporter [57,58], that may help to explain why this open channel blocker has effects on β-cell [Ca^2+^]_i_ and insulin secretion when used in vitro without exogenous agonist/co-agonist, which we found to be critical for NMDAR activation and GSIS effects in the current study.

The inhibitory model of β-cell NMDARs has also been used to explain the reportedly anti-diabetic phenotype of β-cell and pancreatic GluN1 loss models, which our findings support but only to a limited degree. We did find a statistically significant, if small, improvement in glucose tolerance between pGrin1 KO and pGrin1 Het male mice, as previously reported [6], although not compared to Cre- WT controls or in female mice where both pGrin1 heterozygotes and full KOs showed a clear and consistent glucose intolerance. Similarly, while we re-iterated the capacity for βGrin1 KO mice to show a mild improvement in islet GSIS in older animals, there was a much more dramatic secretory impairment in βGrin1 KO islets from younger adult mice. This is not the first murine transgenic β-cell model to show a reversal of insulin secretory phenotype with age (see for example [59]), but it is difficult to know whether this shift represents an age-related change in the developmental role of NMDARs themselves, which has been suggested in other tissues [60], or is a consequence of adaptations undertaken by developing β-cells to cope with the absence of NMDARs since early gestation. An adaptation hypothesis could help explain the normal glucose homeostasis in βGrin1 KO mice, regardless of the status of in vitro GSIS, although CNS effects of a leaky Cre expression on hypothalamic NMDARs [48] could also contribute to an in vivo counter-regulation. Regardless, constitutive-Cre-based GluN1 loss models seem less suited to providing a picture of the endogenous role of β-cell NMDARs than to proving the mechanistic specificity of NMDAR-targeting conditions, as we have done here for the excitatory effects of D-serine/NMDA.

In the absence of a clear and consistent constitutive model, is there evidence for endogenous excitatory D-serine co-agonism of β-cell NMDARs? The inhibitory effects of NMDAR co-agonist site blocker 5,7-DCKA on glucose-stimulated membrane depolarization, and studies implicating NMDAR excitotoxicity in the β-cell response to persistent hyperglycemia [17,21] suggest that elevated glucose may endogenously generate β-cell NMDAR activating conditions. The failure of exogenous D-serine or NMDA alone to augment this effect during in vitro GSIS may simply point to a more even stoichiometry of agonist and co-agonist availability in isolated and perfused islets vs. in vivo. Furthermore, while the data up to this point do not differentiate a specific co-agonist identity, considerable circumstantial evidence points towards D-serine, as opposed to glycine, as the dominant endogenous β-cell co-agonist. Basal pancreatic D-serine has been measured in mice [37] and rats [30,61], while this tissue also expresses all three CNS-characterized D-serine transporter proteins [62], namely ASCT1, ASCT2, and Asc-1 [63,64,65,66]. Moreover, a critical function of D-serine mediated synapses is glycine clearance, as it not only competes for the NMDAR co-agonist binding site, but strongly inhibits Srr [67]. This is primarily achieved by the GlyT1 transporter and small neutral amino acid SNAT transporters, both of which are expressed at the protein level in human and rodent islets/β-cells [66,68]. By contrast, the GlyT2 transporter, which dominates glycinergic synapses in the brain [62], was undetectable in the mouse pancreas [66].

The question of a physiological purpose to D-serine’s modulation of β-cell NMDARs will largely depend on the location and circumstances of D-serine’s release, about which there is, unfortunately, scant data. Both D-serine and glutamate could originate from the blood stream, tying insulin secretion to systemic availability, just as glucose does, possibly through dietary intake or even enterobacterial synthesis [69]. In our previous investigation [1], we observed some serine racemase staining in insulin-negative cells at the periphery of mouse islets, potentially α-cells that could participate in paracrine signaling reminiscent of the astrocytic model of D-serine release proposed in the brain [70]. On the other hand, serine racemase co-localized most strongly with insulin-positive β-cells, which would be consistent with autocrine D-serine signaling, an idea beginning to emerge in neuronal physiology as well. This is an attractive idea, as limited stimulation of β-cell insulin secretion and β-cell D-serine release could potentiate GSIS acutely, a documented phenomenon of positive feedback previously attributed to other autocrine [71] and intracellular [72] mechanisms as well. Additionally, β-cell over-stimulation with repetitive or cumulative D-serine release could prime NMDARs for endocytosis, desensitizing channel modifications and/or trigger non-ionic NMDAR-dependent signaling pathways [73] that act to limit β-cell excitability, serving as a protection against excitotoxicity or diabetogenic exhaustion. Along these lines, prolonged hyperglycemia was noted to significantly increase inhibitory Grin3a transcripts in Min6 β-cells [74], while a SNP in this gene has been associated with β-cell dysfunction and new onset diabetes following kidney transplantation [75,76].

The introduction of GluN3a as a potential variable in NMDAR-dependent outcomes raises some intriguing possibilities. Non-classical, triheteromeric NMDARs (GluN1/GluN2/GluN3a) have two co-agonist binding sites which, in the case of glycine, results in high affinity GluN3a binding and channel activation at low concentration but low affinity GluN1 binding and channel inhibition at high concentration [77], while D-serine acts as a glycine antagonist [78,79] and competitively inhibits the channel [9]. Another surprising quality conferred by GluN3a is a decrease in voltage sensitivity, permitting even tonic NMDAR activation at resting membrane potentials [36] and a dual conductance in which D-serine inhibits these channels at hyperpolarized potentials but activates them during depolarization [9]. So, in addition to the strain-based differences previously discussed, the presence of GluN3a-triheteromeric channels, as a potentially dynamic sub-population of β-cell NMDARs, could contribute to variable concentration and co-agonist identity effects, as well as divergent pharmacological outcomes, at resting vs. stimulated membrane potentials. GluN3a expression is also developmentally regulated, high during the post-natal period and declining with age [36], and responsive to cellular conditioning [36,74], all of which could go a long way towards explaining the multiplicity of in vitro and in vivo outcomes in the published literature. Future investigations will hopefully provide the data to determine which, if any, of these mechanisms contribute to D-serine’s function in islet physiology, including whether the sub-population diversity and context-dependent regulation of NMDARs seen in the CNS is reflected in the pancreas.

## 5. Conclusions

There is not yet a complete picture of all the roles that NMDARs may play in β-cell function. It is not clear, for example, whether NMDAR sub-populations might exist, as they do in the CNS with differing subunit compositions, localization, activation characteristics, function, and purpose, and whether context-based recruitment of these may help to explain the diversity of results produced by various independent investigations. In the current study, we have demonstrated that D-serine acting as an NMDAR co-agonist has the capacity to depolarize β-cells and potentiate islet insulin release, contributing to acute improvements of in vivo glucose tolerance. We have also presented evidence that NMDAR co-agonism, which is likely mediated by D-serine rather than glycine in the pancreas, contributes to the endogenous regulation of β-cell excitability and function but that the parameters of this regulation are highly dependent on context—including background strain, sex, age, and experimental condition. This is perhaps unsurprising given the complexity and range of NMDAR-mediated behaviors characterized in other tissues but does suggest limitations on the translational scope of currently available data and precautions that future investigations carefully consider the choice of an in vivo model and in vitro environment to insure mechanistic specificity in the interpretation of outcome.

## 6. Patents

AL is the author of a patent (US 9,339,482), owned by the University of Minnesota and funded by its Office of Technology Commercialization, which describes the potential therapeutic use of D-serine and other NMDAR co-agonist site drugs for treating dysregulated blood glucose disorders.

## Data Availability

Data is contained within the article or supplementary material.

## Supplementary Materials

The following are available online at https://www.mdpi.com/2073-4409/10/1/93/s1, Figure S1: DAAO−/− Serum D-serine; Figure S2: D-serine Effects in Multiple Mouse Strains, Figure S3: Strain Differences in Grin3a Expression; Figure S4: Dser/NMDA response in WT Dispersed β-cells, Figure S5: Dser/NMDA Response in βGrin1 KO Dispersed β-cells, Figure S6: βGrin1 KO Male High Fat Diet Phenotype, Figure S7: βGrin1 KO Female High Fat Diet Phenotype, Figure S8: Pancreatic Cre-GFP Expression.
