# Supplementary material for: Acute D-Serine Co-Agonism of β-Cell NMDA Receptors Potentiates Glucose-Stimulated Insulin Secretion and Excitatory β-Cell Membrane Activity
Source: Cells. 2021 Jan 7;10(1):93. doi: 10.3390/cells10010093 (PMC7826616; doi:10.3390/cells10010093)
Supplement: Supplementary file 1 [file cells-10-00093-s001.pdf]

SFig 1. DAAO$^{-/-}$ Serum D-serine

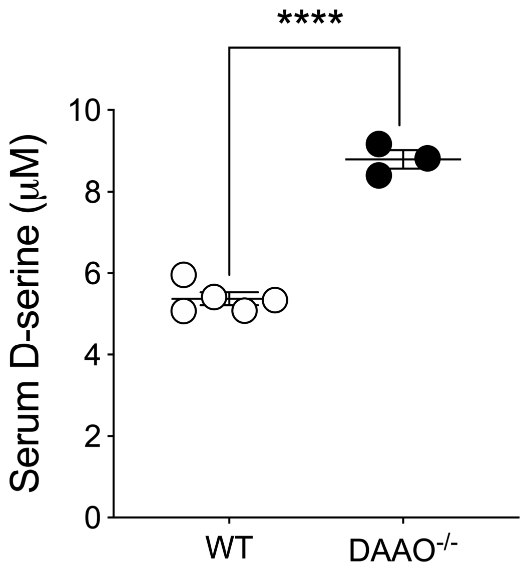

Supplemental Figure 1. Serum D-serine in DAAO mutant mice. Related to figure 1. Preliminary data shows serum D-serine levels from ~70-day-old DAAO$^{-/-}$ and ddY WT mice, as detected by capillary electrophoresis. Sex of the mice was not noted at the time of harvest. Statistical analysis was by independent 2-tailed t-test. ****$p < 0.00001$.

SFig 2. D-serine Effects in Multiple Mouse Strains

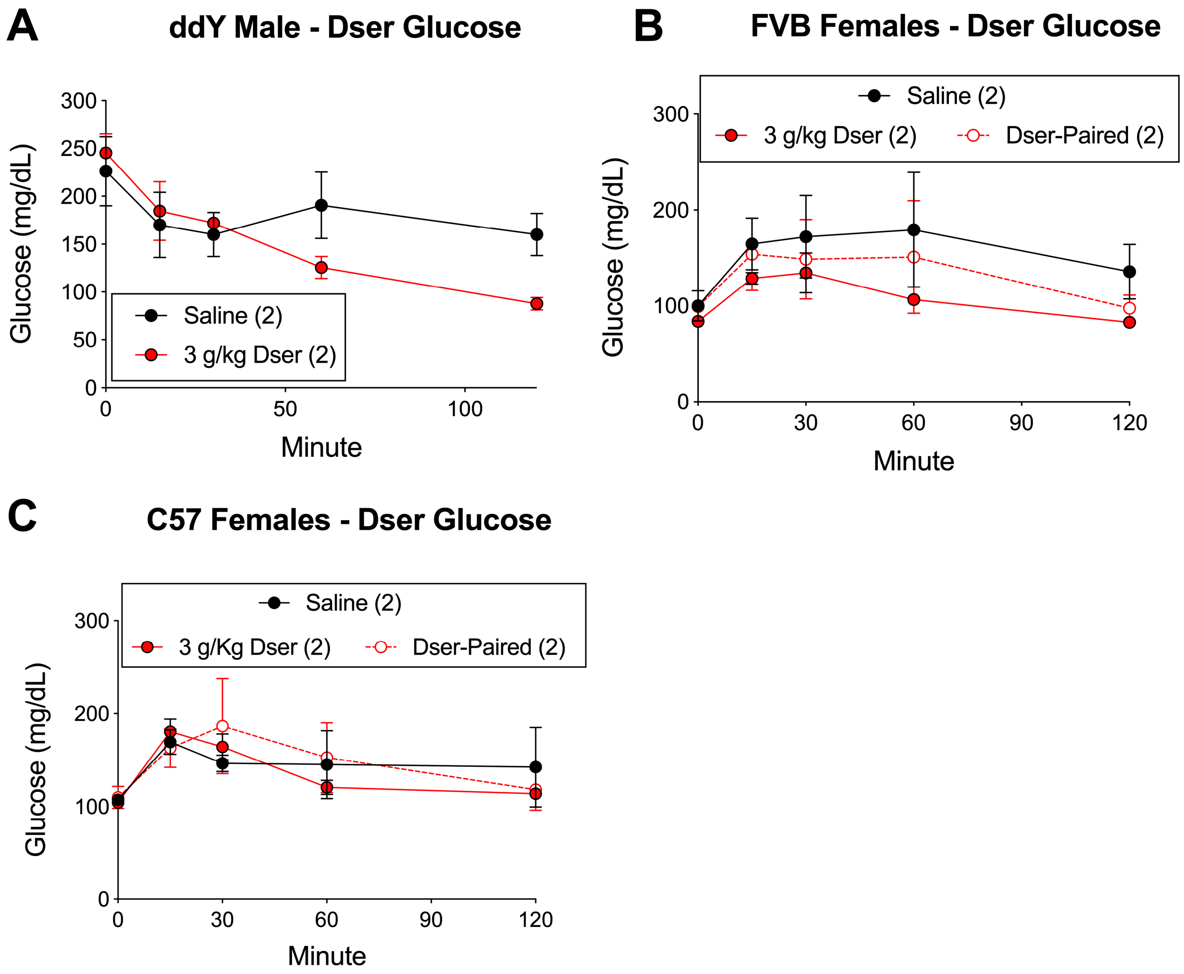

Supplemental Figure 2. D-serine hypoglycemic response in multiple mouse strains. Related to figure 1. As part of a preliminary assessment of D-serine's acute effect, tail vein blood glucose was monitored up to 120 min after an i.p. injection of D-serine (Dser, 3 g/kg) or saline in randomly-fed mice from (**A**) ddY strain males, (**B**) FVB strain females and (**C**) C57 strain females. Dser-Paired shows the average D-serine-response of the saline control mice, re-tested after a 3-day rest period. N numbers are listed in the legend. Statistical analysis was not performed due to the small group size.

SFig 3. Strain Differences in Grin3a Expression

A

Hypothalamic Expression of D-serine Related Genes (TPM)

Raw data from Zhang, 2020

	C57Bl/6J				FVB/NJ			
	Mouse 1	Mouse 2	Mouse 3	Mouse 4	Mouse 1	Mouse 2	Mouse 3	Mouse 4
**Grin1**	45.67	43.58	41.14	43.93	40.79	45.51	29.22	33.00
**Grin2a**	10.63	8.84	8.45	6.95	8.59	7.70	4.58	7.15
**Grin2b**	15.63	8.74	12.48	10.39	13.46	10.20	4.51	9.15
**Grin2c**	11.63	10.06	10.27	8.07	9.09	10.96	6.69	8.66
**Grin2d**	2.78	1.72	3.18	2.80	2.98	3.15	1.49	2.19
**Grin3a**	13.32	12.97	12.40	13.34	11.85	10.99	6.00	10.53
**Grin3b**	0.20	0.17	0.26	0.27	0.20	0.26	0.12	0.15
**Srr**	10.24	10.82	9.18	10.42	8.64	7.88	6.16	8.36
**Dao**	0.09	0.13	0.13	0.06	0.06	0.18	0.05	0.06

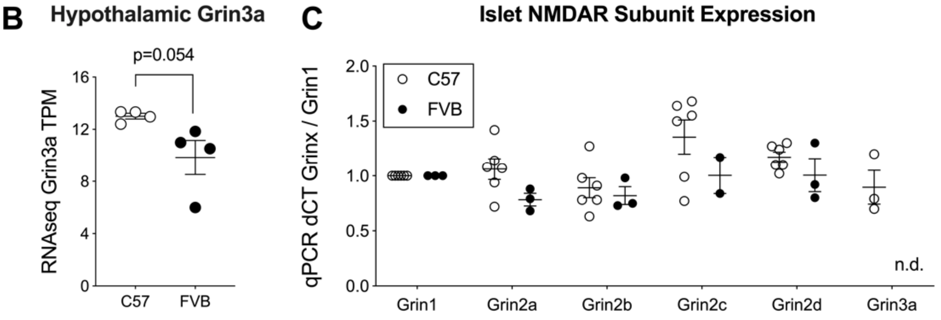

D

GluN3a Staining - C57 Pancreas

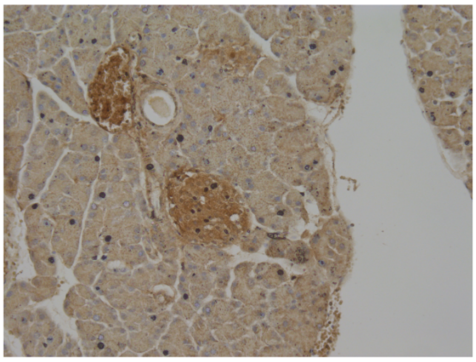

Supplemental Figure 3. D-serine related gene expression in C57 and FVB strains. Related to figures 1 and 2. To evaluate strain differences in D-serine relevant genes, TPM (transcripts per kilobase million) data for NMDAR subunits (Grin1-3) and D-serine synthetic (Srr) and catabolic (Dao) enzymes was extracted from the RNAseq files uploaded to the Genome Expression Omnibus (GEO) database by Zhang *et al.* (PMID: 31669422, GSE123893). (**A**) Candidate gene expression from the hypothalamus of water-drinking C57Bl/6J and FVB/NJ samples was averaged over 3 replicates per mouse (except 2 instances of majority 0 coverage) with highlights indicating near significant comparisons ($p \leq 0.05$ by independent 2-tailed t-test) and (**B**) Grin3a is also displayed in a scatter plot. (**C**) From the current study, qPCR data from isolated islets of both strains (N = 6 C57, N = 4 FVB) shows expression of NMDAR subunits relative to the requisite

receptor forming Grin1 encoded subunit (dCT Grin*x*/dCT Grin1, per mouse). (**D**) An example of GluN3a immunohistochemical staining in a C57 fixed pancreas section. n.d. = not detected.

SFig 4. Dser/NMDA Response in WT Dispersed β-cells

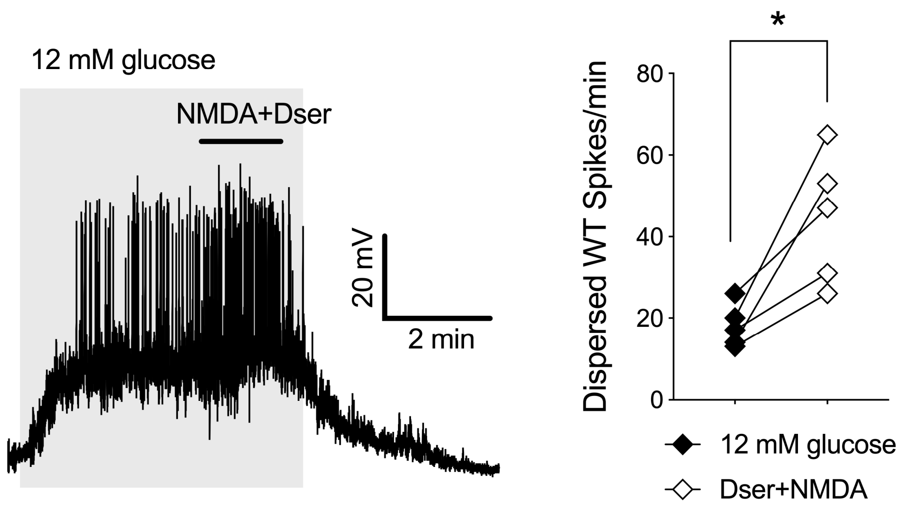

Supplemental Figure 4. Dser/NMDA Response in WT dispersed β-cells. Related to figure 2. Glass-plated large diameter single cells, derived from trypsin-digested primary C57 mouse islets, were targeted for perforated-patch recording. After establishing β-cell identity through an excitatory Vm response to a switch from 3 mM to 12 mM glucose (grey box), cells were bathed in external D-serine and NMDA (100 uM each) and then allowed to return to baseline in 3 mM glucose (left). Spike rate was quantified during the 12 mM glucose and glucose + Dser/NMDA conditions for each cell (right, $n = 5$). Statistical analysis was by 2-tailed paired t-test. $^*p < 0.05$ as indicated.

SFig 5. Dser/NMDA Response in βGrin1 KO Dispersed β-cells

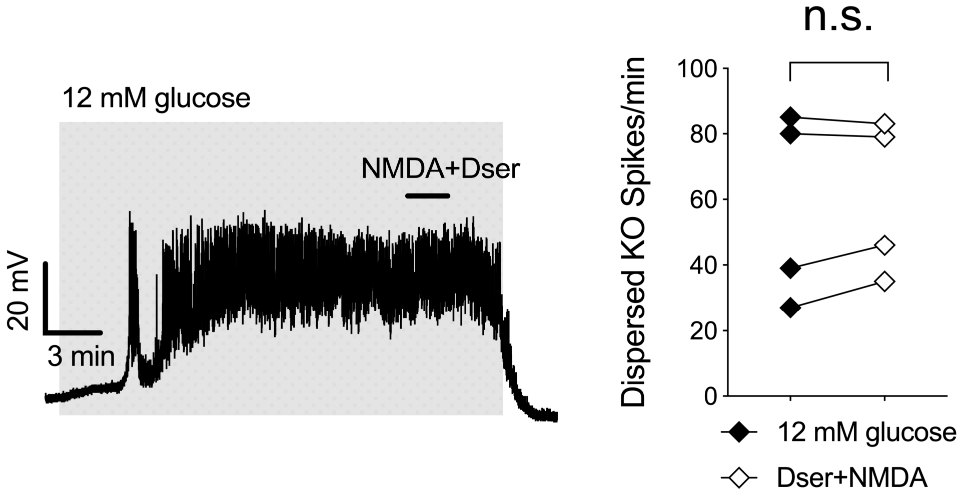

Supplemental Figure 5. Dser/NMDA response in Grin1 KO dispersed β-cells. Related to figure 3. Excitatory membrane responses to glucose and Dser/NMDA were recorded from dispersed β-cells, described in SFig4, derived from C57 mice with a β-cell specific genetic deletion of the requisite subunit of the NMDAR channel. A representative trace is shown on the left and a quantification of spike rate on the right (*n* = 4). Statistical analysis was by 2-tailed paired t-test. Not significant (n.s.) $p > 0.05$ as indicated.

SFig 6. βGrin1 KO Male High Fat Diet Phenotype

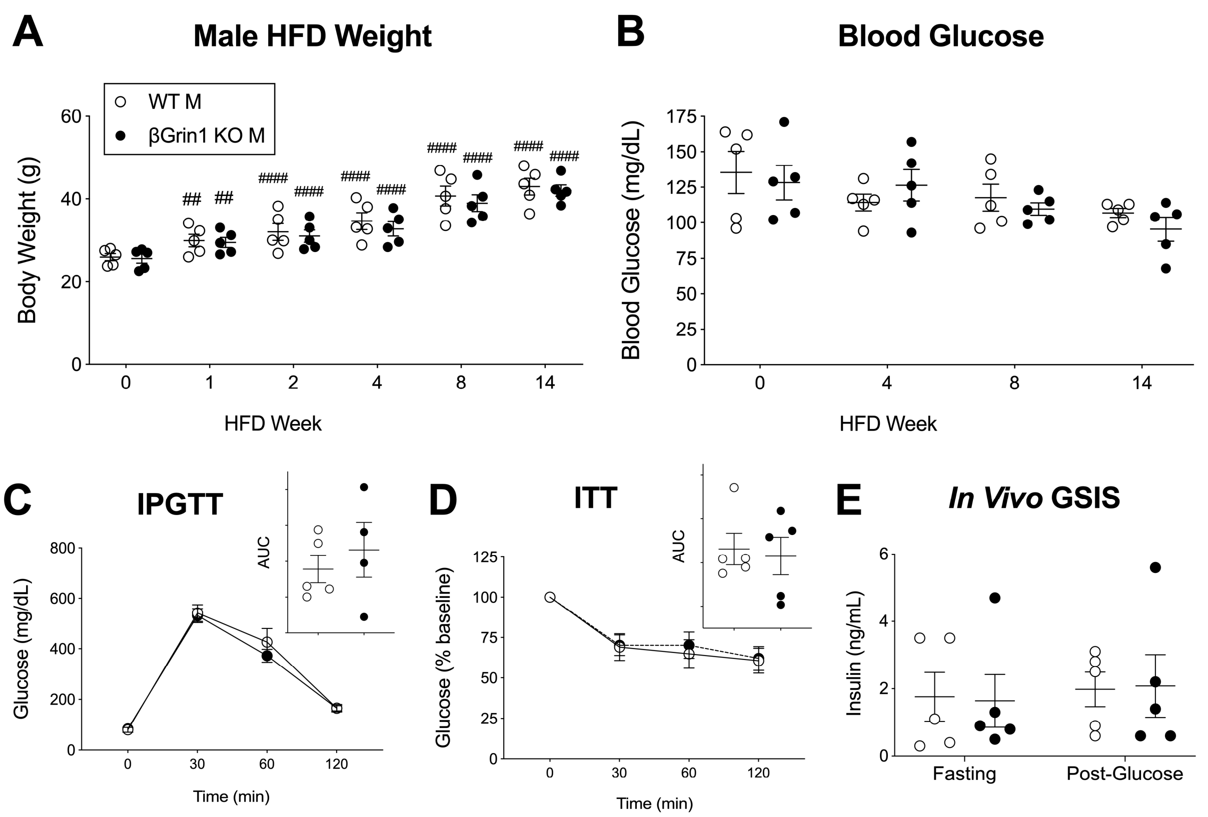

Supplemental Figure 6. Diet-induced obesity in βGrin1 KO male mice. Related to figure 4. 3-to-4-month old male WT (white) and βGrin1 KO (black) mice were given up to 14 weeks of *ad libitum* access to high fat diet (HFD, 60% kcal fat) and evaluated for glucose homeostasis and insulin secretory measures. (**A**) Random fed body weight and (**B**) blood glucose are shown at progressive weeks of HFD on the x-axis. (**C**) Mice were tested for glucose tolerance in response to 2 g/kg ip glucose after overnight fasting at 8 wks HFD and (**D**) insulin sensitivity in response to 0.75 U/kg ip insulin after 6-hr fasting at 6 wks HFD. (**E**) *In vivo* GSIS shows overnight fasted and glucose-stimulated (3 g/kg ip) insulin secretion in mice after 14 wks HFD feeding. Area under the curve is shown in the inset for panels C and D. All experiments were analyzed by repeated measures 2-way ANOVA. ##$p < 0.01$, ####$p < 0.0001$ vs. w/in genotype Week 0. ITT = insulin tolerance test. Figure legend in panel A defines all symbols.

SFig 7. βGrin1 KO Female High Fat Diet Phenotype

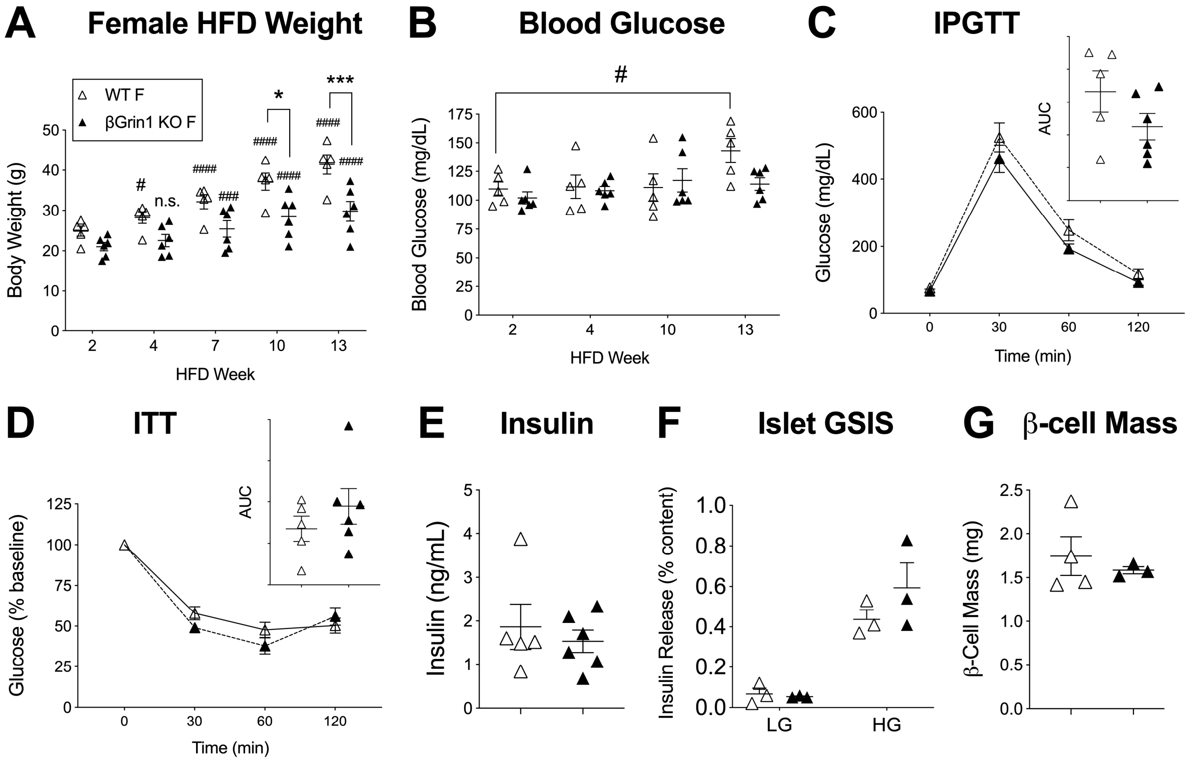

Supplemental Figure 7. Diet-induced obesity in βGrin1 KO female mice. Related to figure 4. 3-month-old female WT (white) and βGrin1 KO (black) mice were given up to 13 weeks of *ad libitum* HFD. Similar to SFig 6, mice were assessed for (**A**) body weight and (**B**) blood glucose at 2–3 weeks intervals, (**C**) glucose tolerance at 7 wks HFD, (**D**) insulin sensitivity at 5 wks HFD and (**E**) random-fed plasma insulin at 13 wks HFD. (**F**) Islets were isolated from some mice after 13 wks to assess insulin secretion in response to low glucose (LG, 2 mM) and high glucose (HG, 22 mM) stimulation and (**G**) pancreata were harvested from others and fixed after 15 wks HFD for evaluation of β-cell mass. Area under the curve is shown in the inset for panels C and D. Insulin and β-cell mass were analyzed by 2-tailed t-test and all other experiments by repeated measures 2-way ANOVA with Sidak's multiple comparisons. *$p < 0.05$, ***$p < 0.001$ vs. WT. n.s. $p > 0.05$, #$p < 0.05$, ###$p < 0.001$, ####$p < 0.0001$ vs. Week 2. Figure legend in panel A defines all symbols. LG = low glucose, 2 mM. HG = high glucose, 22 mM.

SFig 8. Pancreatic Cre-GFP expression

Pdx-cre; Grin1 f/f; CAG +/-

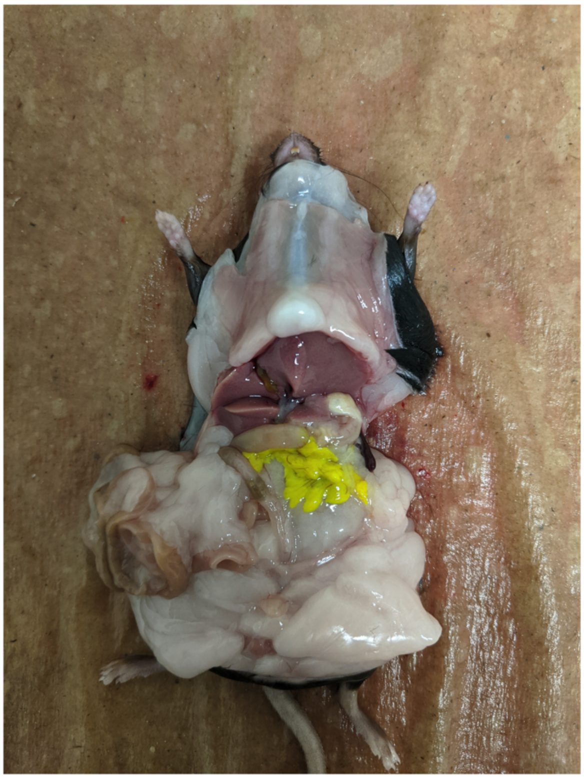

Supplemental Figure 8. Pancreatic Cre-GFP expression. Related to figure 6. Picture of pancreatic ZsGreen expression in a Pdx-cre; Grin1 f/f; $CAG^{+/-}$ (pGrin1 KO) female mouse. CAG-ZsGreen was bred into the mouse colony to serve as a tissue reporter of Cre expression.
